# Clinical Manifestations and Outcomes of Ocular Graft Versus Host Disease following Allogeneic Stem Cell Transplantation

**DOI:** 10.18502/jovr.v19i3.13095

**Published:** 2024-09-16

**Authors:** Vijay Shetty, Priyanka Kashelkar, Sachin Punatar, Suhas Haldipurkar, Abhishek Hoshing, Rasika Thakur, Prachi Sankhe, Shabnam Tanwar, Tanvi Haldipurkar, Maninder Singh Setia, Anant Gokarn, Lingaraj Nayak, Avinash Bonda, Navin Khattry

**Affiliations:** ^1^Laxmi Eye Institute, Panvel, Navi Mumbai, India; ^2^Stem Cell Transplant Unit, Department of Medical Oncology, ACTREC, Tata Memorial Centre, Navi Mumbai, India; ^3^Homi Bhabha National Institute (HBNI), Mumbai, India; ^5^Vijay Shetty: https://orcid.org/0000-0003-0544-0200; ^6^Maninder Singh Setia: https://orcid.org/0000-0003-1291-9033

**Keywords:** Anterior Segment, Cornea, Longitudinal Study, Ocular Graft-versus-Host Disease, Posterior Segment

## Abstract

**Purpose:**

To evaluate clinical presentation of chronic ocular graft-versus-host disease (GVHD), laterality of presentation, and longitudinal changes in patients undergoing allogeneic stem cell transplantation.

**Methods:**

This is a retrospective longitudinal analysis of 60 eyes from 30 patients who had undergone hematopoietic stem cell transplantation. Demographic characteristics, clinical history, comorbidities, and other organ involvements were taken into account for analysis. We also undertook complete evaluation of the eyes, including cornea and anterior segment, posterior segment, Schirmer test, tear break-up time, ocular surface disease index, and intraocular pressure.

**Results:**

The mean age of the patients was 34.3 ± 11 years. The mean time for the diagnosis of ocular GVHD was 232.8 days (95% CI: 153.6, 311.9). The common findings at the first visit were bilateral blepharitis (*n* = 5, 17%), meibomitis (*n* = 4, 13%), and conjunctival congestion (*n* = 3, 10%). While bilateral cataract was present in one (3%) patient at the first visit, at 18 months, five (17%) patients had bilateral cataract and one (3%) patient had unilateral cataract. Grade 1 (*n* = 17), grade 2 (*n* = 9), and grade 3 (*n* = 4) superficial punctate epithelial erosions (SPEEs) were also observed at the first visit. However, SPEEs were seen in only 11 eyes at 18 months; all of these cases were grade 1 SPEEs. Long-term findings included cataract, telangiectasia, blepharospasm, conjunctival congestion, grade 1 SPEEs, corneal filaments, and tear film debris.

**Conclusion:**

Although the initial presentations were SPEEs, meibomitis, blepharitis, and conjunctival congestion, these inflammatory conditions were reduced over time with proper management. However, there was an increase in the proportion of patients with cataract. It is important to regularly monitor these patients in order to identify and manage the initial as well as the late ocular manifestations of chronic GVHD.

##  INTRODUCTION

Hematopoietic stem cell transplantation (HSCT) is useful for management of hematological malignancies, several benign hematological disorders, and metabolic conditions; however, its long-term success is hampered by the occurrence of graft-versus-host disease (GVHD).^[1,2]^ The estimated incidence of acute GVHD is about 50%, and the incidence of chronic GVHD may vary from 6% to 80%.^[3]^ GVHD occurs when the donor T-cells attack the host tissues. The risk factors for GVHD include old age of the donor or recipient, gender of the donor (female gender), mismatch between the donor and recipient human leukocyte antigens (HLAs), use of peripheral blood stem cells, etc.^[3,4]^

Conventionally, GVHD was considered acute if it occurred before 100 days of transplant, and chronic if it occurred after 100 days of transplant.^[1,2]^ However, acute GVHD can emerge after 100 days (late-onset acute GVHD), and there could be overlapping features between acute and chronic GVHD (overlap syndromes).^[3]^ A recent report by the National Institutes of Health (NIH) focused on clinical features, rather than the time of presentation, in classifying GVHD as either acute or chronic.^[5]^ Corticosteroids along with calcineurin inhibitors are used for initial management of acute and chronic GVHD.^[6]^ Some other agents administered to manage acute and chronic GVHD include monoclonal antibodies, anti-interleukin-2 receptor antibodies, anti-tumor necrosis factor alpha agents, mycophenolate mofetil, sirolimus, and extracorporeal photopheresis.^[6]^

Ocular GVHD (oGVHD) may occur in about 60% of cases of allogenic HSCT and has a wide range of clinical manifestations.^[7,8,9]^ Presence of systemic GVHD, male recipient (of female grafts), and certain comorbidities (such as diabetes) are associated with a higher occurrence of oGVHD, whereas race (Caucasians) is associated with a lower occurrence of oGVHD.^[7,8]^ Although GVHD may involve all layers of the eye, the common clinical manifestations of oGVHD in the acute phase are corneal epithelial keratitis or conjunctivitis (hemorrhagic or a less severe form).^[1][7,8][10]^ Chronic oGVHD may show features of corneal epithelial erosions, keratitis, conjunctival involvement, and meibomian and lacrimal gland involvement.^[8]^ Involvement of the posterior segment, however, is not common.^[1]^ Due to its adverse effects on the eye, oGVHD has a negative impact on the quality of life of patients with this condition.^[11]^

Previous studies have highlighted the prevalence and incidence of various manifestations in acute and chronic oGVHD.^[12,13,14]^ In this study, we evaluated the clinical presentation of chronic oGVHD, laterality of presentation, and changes in these presentations over time in patients who had undergone allogeneic stem cell transplantation.

##  METHODS

The present study is a retrospective analysis of clinical data of 60 eyes from 30 patients admitted to a tertiary eye care center in Panvel, India. The study was approved by the Institutional Ethics Committee (Reference No. LEI/003/2016 Amendment dated February 01, 2023).

### Study Participants, Data, and Diagnostic Criteria

All patients who had undergone stem cell transplantation and were referred to our center post-HSCT between January 2011 and April 2014 were included for the present analysis. We reviewed the records of patients diagnosed with oGVHD and initiated their specialized ophthalmological treatment. The following information was retrieved from the records:

(1) Demographic characteristics; (2) Clinical history (presenting complaints, indication for HSCT, and comorbidities); and (3) Other organ involvement (liver, gastrointestinal tract, oral cavity, lungs, and skin).

We examined laboratory parameters such as white blood cell count, platelet count, and bilirubin levels on day 1 and day 100. We also collected treatment information, including local therapy as well as the use of systemic immunosuppressants, antifungals, antibiotics, antivirals, and steroids.

We used the NIH criteria (2014) for the diagnosis of chronic GVHD and oGVHD. The criteria for chronic GVHD as recommended by the NIH Working Group were as follows:

(1) At least one diagnostic manifestation of chronic GVHD; (2) At least one distinctive manifestation and related laboratory or other tests such as pulmonary function tests and Schirmer test; (3) Evaluation by specialists such as an ophthalmologist; and (4) Radiological evaluation showing chronic GVHD in the same or other organs.^[15]^

Similarly, the NIH criteria (2014) for the diagnosis of oGVHD that we considered in the present study were as follows:

(1) Distinctive signs of new ocular sicca with a Schirmer test value <5 mm/5 min (preferable with confirmation of normal values at an established baseline); or (2) New onset of keratoconjunctivitis sicca identified by slit lamp examination with a mean Schirmer test value between 6 and 10 mm/5 min (preferable with confirmation of normal values at an established baseline); and (3) Additional distinctive feature of at least another organ involvement.^[15]^

**Table 1:** Table showing the anterior segment manifestations in 60 eyes of 30 patients who had undergone stem cell transplantation, Panvel, India ${\;}^{*}$ .


**Anterior segment manifestations**	**Visit 1**	**Visit 2**	**Visit 3**	**Visit 4**	**Visit 5**	**Visit 6**	**Visit 7**
	**Eye**	**Initial visit**	**6 months**	**12 months**	**18 months**	** > 24 months**	** > 24 months**	** > 24 months**
		*n* (%)	*n* (%)	*n* (%)	*n* (%)	*n* (%)	*n* (%)	*n* (%)
Within normal limits	Bilateral	13 (43)	10 (33)	10 (33)	6 (20)	1 (3)	1 (3)	0 (0)
	Unilateral	1 (3)	1 (3)	0 (0)	0 (0)	0 (0)	0 (0)	0 (0)
Discharge	Bilateral	2 (7)	0 (0)	0 (0)	0 (0)	0 (0)	0 (0)	0 (0)
	Unilateral	0 (0)	0 (0)	0 (0)	0 (0)	1 (3)	0 (0)	0 (0)
Blepharitis	Bilateral	5 (17)	2 (7)	3 (10)	2 (7)	1 (3)	0 (0)	0 (0)
	Unilateral	0 (0)	1 (3)	0 (0)	0 (0)	1 (3)	0 (0)	0 (0)
Meibomitis	Bilateral	4 (13)	9 (30)	6 (20)	3 (10)	5 (17)	1 (3)	2 (7)
	Unilateral	0 (0)	0 (0)	0 (0)	0 (0)	0 (0)	1 (3)	0 (0)
Uveitis	Bilateral	1 (3)	0 (0)	0 (0)	0 (0)	0 (0)	0 (0)	0 (0)
	Unilateral	1 (3)	0 (0)	0 (0)	0 (0)	0 (0)	0 (0)	0 (0)
Blepharospasm	Bilateral	0 (0)	1 (3)	1 (3)	0 (0)	1 (3)	0 (0)	0 (0)
	Unilateral	1 (3)	0 (0)	0 (0)	0 (0)	1 (3)	1 (3)	0 (0)
Conjunctival congestion	Bilateral	3 (10)	0 (0)	1 (3)	0 (0)	2 (7)	1 (3)	0 (0)
	Unilateral	0 (0)	0 (0)	0 (0)	0 (0)	0 (0)	0 (0)	0 (0)
Cataract	Bilateral	1 (3)	3 (10)	3 (10)	5 (17)	5 (17)	2 (7)	1 (3)
	Unilateral	0 (0)	1 (3)	0 (0)	1 (3)	3 (10)	2 (7)	1 (3)
Follicles	Bilateral	0 (0)	2 (7)	0 (0)	0 (0)	0 (0)	0 (0)	0 (0)
	Unilateral	0 (0)	1 (3)	0 (0)	0 (0)	1 (3)	0 (0)	0 (0)
Telangiectasia	Bilateral	0 (0)	0 (0)	0 (0)	0 (0)	1 (3)	2 (7)	2 (7)
	Unilateral	0 (0)	1 (3)	2 (7)	0 (0)	0 (0)	0 (0)	0 (0)
Keratinized lid	Bilateral	0 (0)	1 (3)	0 (0)	0 (0)	1 (3)	0 (0)	0 (0)
	Unilateral	0 (0)	0 (0)	0 (0)	0 (0)	0 (0)	0 (0)	0 (0)
	
	
${\;}^{*}$ The proportions are based on the total sample size								

**Table 2:** Table showing the posterior segment manifestations in 60 eyes of 30 patients who had undergone stem cell transplantation, Panvel, India ${\;}^{*}$ .


**Posterior segment manifestations**	**Visit 1**	**Visit 2**	**Visit 3**	**Visit 4**	**Visit 5**	**Visit 6**	**Visit 7**
	>**Eye**	**Initial visit**	**6 months**	**12 months**	**18 months**	** > 24 months**	** > 24 months**	** > 24 months**
		*n* (%)	*n* (%)	*n* (%)	*n* (%)	*n* (%)	*n* (%)	*n* (%)
Within normal limits	Unilateral	3 (10)	1 (3)	1 (3)	1 (3)	0 (0)	0 (0)	0 (0)
	Bilateral	23 (77)	26 (87)	23 (77)	16 (53)	12 (40)	8 (27)	5 (17)
Uveitis	Unilateral	1 (3)	0 (0)	0 (0)	0 (0)	0 (0)	0 (0)	0 (0)
	Bilateral	0 (0)	0 (0)	0 (0)	0 (0)	0 (0)	0 (0)	0 (0)
RPE changes	Unilateral	1 (3)	0 (0)	0 (0)	0 (0)	0 (0)	0 (0)	0 (0)
	Bilateral	0 (0)	0 (0)	0 (0)	0 (0)	0 (0)	0 (0)	0 (0)
Glaucoma	Unilateral	1 (3)	1 (3)	0 (0)	0 (0)	0 (0)	0 (0)	0 (0)
	Bilateral	0 (0)	0 (0)	0 (0)	0 (0)	0 (0)	0 (0)	0 (0)
White choroidal patch	Unilateral	1 (3)	1 (3)	1 (3)	1 (3)	0 (0)	0 (0)	0 (0)
	Bilateral	0 (0)	0 (0)	0 (0)	0 (0)	0 (0)	0 (0)	0 (0)
	
	
${\;}^{*}$ The proportions are based on the total sample size RPE, retinal pigment epithelium								

**Table 3:** Table showing the corneal manifestations in 60 eyes of 30 patients who had undergone stem cell transplantation, Panvel, India ${\;}^{*}$ .


**Corneal Manifestations**	**Visit 1**	**Visit 2**	**Visit 3**	**Visit 4**	**Visit 5**	**Visit 6**	**Visit 7**
	**Eye**	**Initial visit**	**6 months**	**12 months**	**18 months**	** > 24 months**	** > 24 months**	** > 24 months**
		*n* (%)	*n* (%)	*n* (%)	*n* (%)	*n* (%)	*n* (%)	*n* (%)
Clear	Unilateral	4 (13)	2 (7)	4 (13)	5 (17)	3 (10)	2 (7)	1 (3)
	Bilateral	7 (23)	4 (13)	7 (23)	4 (13)	3 (10)	1 (3)	2 (7)
SPEE Grade 1	Unilateral	3 (10)	1 (3)	13 (10)	3 (10)	3 (10)	3 (10)	1 (3)
	Bilateral	6 (20)	3 (10)	8 (27)	4 (13)	3 (10)	3 (10)	2 (7)
SPEE Grade 2	Unilateral	0 (0)	2 (7)	0 (0)	0 (0)	0 (0)	0 (0)	0 (0)
	Bilateral	4 (13)	6 (20)	0 (0)	0 (0)	0 (0)	0 (0)	0 (0)
SPEE Grade 3	Unilateral	2 (7)	1 (3)	0 (0)	0 (0)	0 (0)	0 (0)	0 (0)
	Bilateral	1 (3)	0 (0)	1 (3)	0 (0)	0 (0)	0 (0)	0 (0)
Filaments	Unilateral	0 (0)	2 (7)	1 (3)	2 (7)	2 (7)	2 (7)	1 (3)
	Bilateral	1 (3)	1 (3)	1 (3)	1 (3)	0 (0)	0 (0)	0 (0)
Tear film defect	Unilateral	1 (3)	0 (0)	0 (0)	0 (0)	0 (0)	0 (0)	0 (0)
	Bilateral	0 (0)	1 (3)	0 (0)	0 (0)	0 (0)	0 (0)	0 (0)
Keratic precipitates	Unilateral	1 (3)	0 (0)	0 (0)	0 (0)	0 (0)	0 (0)	0 (0)
	Bilateral	1 (3)	1 (3)	0 (0)	0 (0)	0 (0)	0 (0)	0 (0)
Tear film debris	Unilateral	0 (0)	0 (0)	0 (0)	0 (0)	0 (0)	0 (0)	0 (0)
	Bilateral	0 (0)	1 (3)	0 (0)	0 (0)	1 (3)	1 (3)	0 (0)
Epithelial defect	Unilateral	0 (0)	0 (0)	0 (0)	0 (0)	0 (0)	0 (0)	0 (0)
	Bilateral	0 (0)	0 (0)	0 (0)	0 (0)	0 (0)	0 (0)	0 (0)
	
	
${\;}^{*}$ The proportions are based on the total sample size SPEE, superficial punctate epithelial erosions								

**Figure 1:**
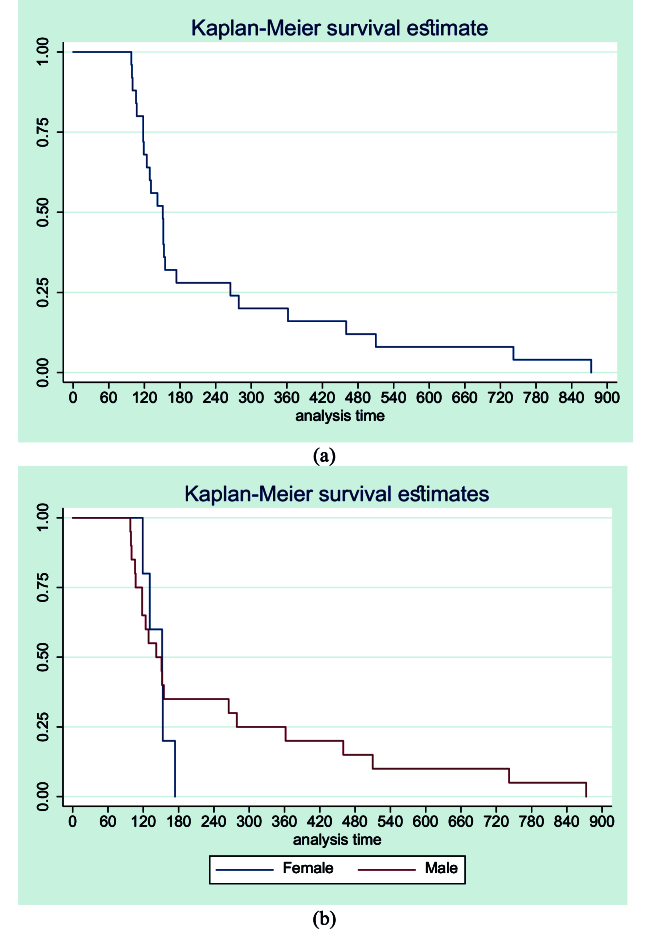
(a) Kaplan-Meier curve showing the time to diagnosis of ocular graft versus host disease. (b) Kaplan-Meier curve showing the time to diagnosis of ocular graft versus host disease according to gender.

**Figure 2:**
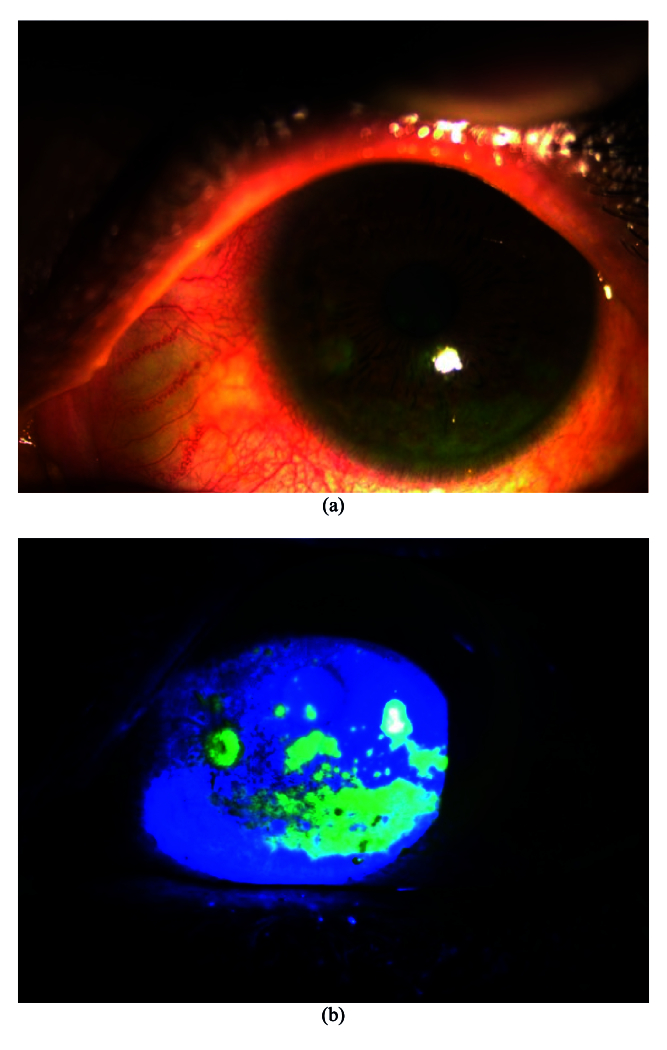
(a) Image of left eye cornea with inferior superficial punctate epithelial erosions (SPEEs) and bulbar conjunctival congestion under diffuse slit lamp illumination. (b) Image showing fluorescein staining of SPEEs on cornea of same eye.

**Figure 3:**
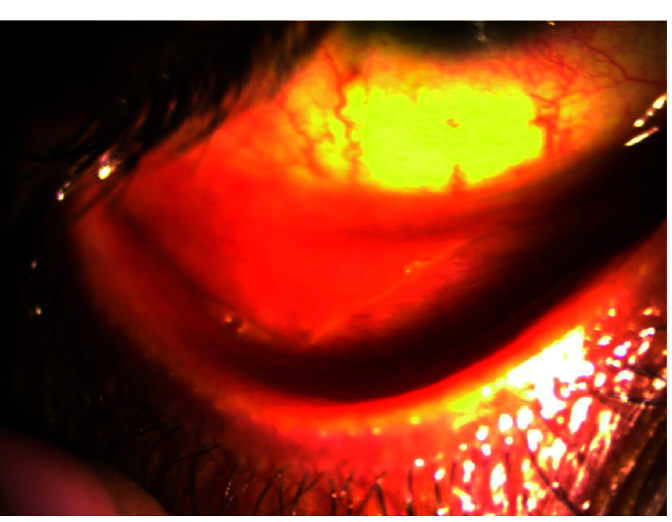
Image showing right eye with meibomian gland dysfunction and palpebral conjunctival congestion on high magnification under diffuse slit lamp illumination.

**Figure 4:**
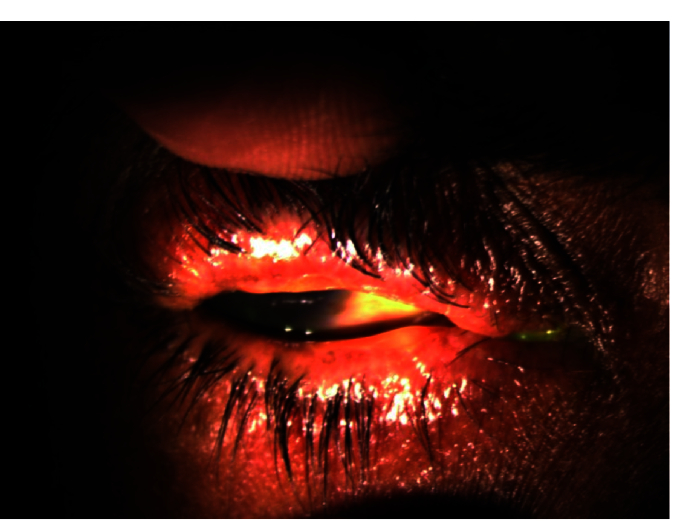
Image showing right eye with thickening and keratinization of lid margin in severe dry eye disease under diffuse slit lamp illumination.

**Figure 5:**
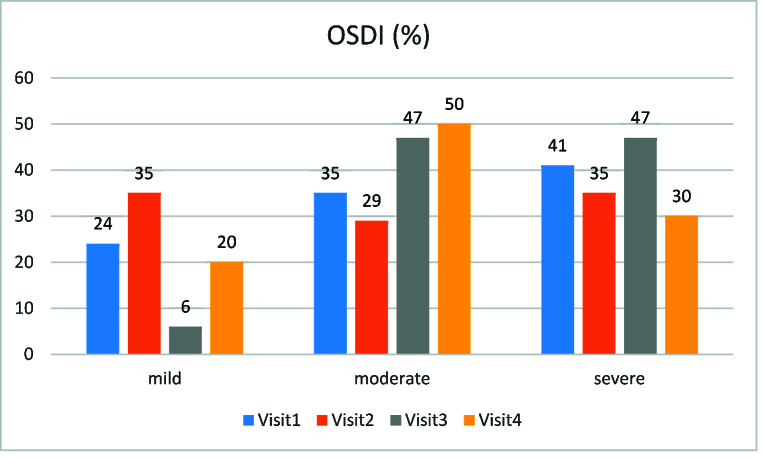
Bar diagram showing the proportion according to ocular surface disease index questionnaire.

**Figure 6:**
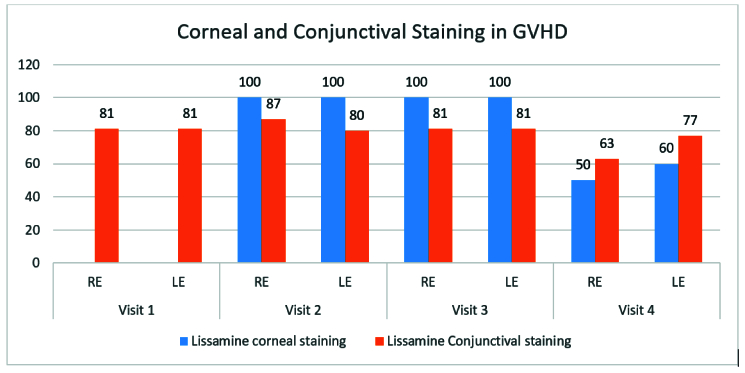
Bar diagram showing the proportion of corneal and conjunctival lissamine staining. LE, left eye; RE, right eye.

**Figure 7:**
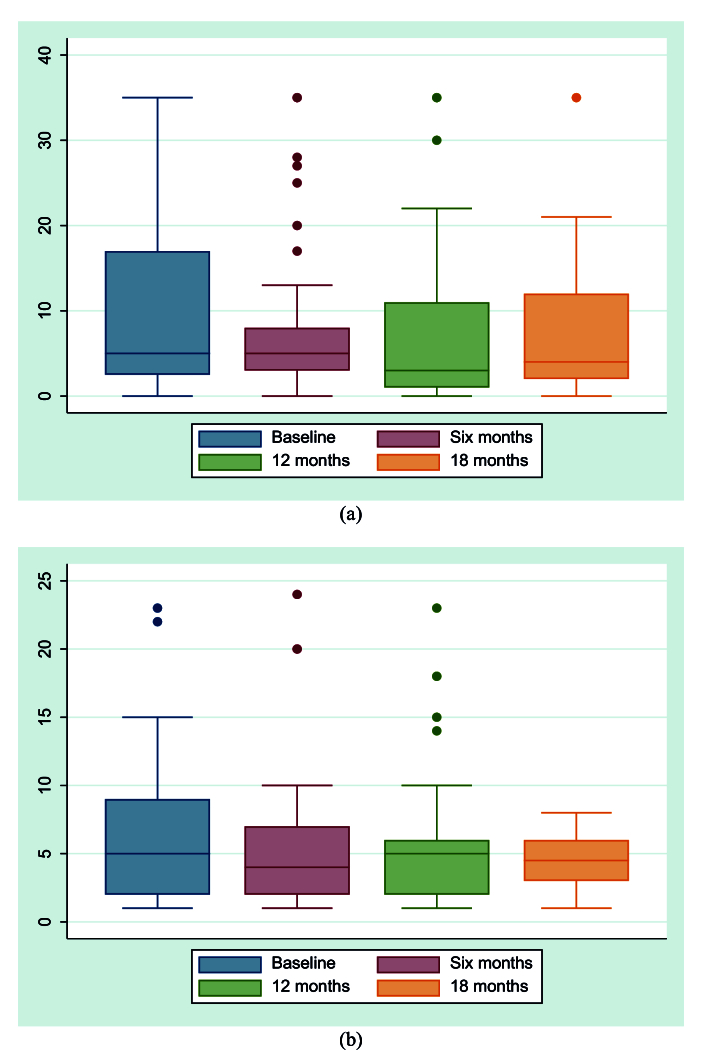
(a) Box diagram showing the values for Schirmer's test. (b) Box plot showing the values for tear break-up time.

### Examination of the Eye

We recorded the presenting symptoms at each visit and conducted detailed clinical assessment, including surface staining and slit-lamp examination. Detailed history of the topical and oral medications was also taken at each visit. The follow-up visits were scheduled at 6 months (+/15 days), 12 months (+/–30 days), and 18 months (+/–30 days). We also recorded data from three visits after completion of 24 months from all patients who were followed-up regularly (the data were last recorded in December 2021). Besides, we performed the following tests:

(1) Corrected distance visual acuity using Snellen’s visual acuity chart at a distance of 6 m; (2) Schirmer test using sterile paper strips (recorded after 5 min); (3) Tear film break-up time (TBUT) using the slit lamp after staining the cornea with sodium fluorescein (recorded in seconds); (4) Lissamine green staining test using 1% lissamine green to stain the cornea and conjunctiva (the result was evaluated under low illumination after 1–4 min); (5) Ocular surface disease index (OSDI) for dry eyes; (6) Detailed slit-lamp examination of the anterior segment to examine the adnexa, lids, cornea, anterior chamber, iris, and lens; (7) Posterior segment examination using a 90- diopter (90D) lens; (8) Intraocular pressure measurement using an applanation tonometer; and (9) Detailed glaucoma evaluation (gonioscopy and perimetry) using Humphrey Field Analyzer.

Local and systemic treatment received by the patient at each visit was also noted. We used the National Eye Institute/Industry grading system to grade the corneal and conjunctival staining.^[16]^ Staining was classified from Grade 0 to Grade 3 based on the density scale. We used the Japanese criteria for the diagnosis of dry eye, which are as follows:

(1) Assessing subjective symptoms; (2) Tear function tests, including Schirmer test <5 mm (5 min), TBUT < 5 s; and (3) Vital staining, comprising lissamine green, rose bengal, and fluorescein stain.^[17,18]^

The patients were treated with a combination of topical steroids, antibiotics, and lubricants. Autologous serum was used to treat a few severe cases. Depending on the severity of the signs and symptoms, the type and frequency of topical steroids were determined and were tapered according to the clinical response. The lubricants were given for symptomatic relief, and the type and frequency of lubricants were also determined based on clinical presentation.

### Statistical Analysis

We estimated the mean and standard deviation for continuous variables and proportion for categorical variables. The means were compared using the t-test and the proportions were compared using the Chi-square test or Fisher exact test for low expected cell counts. We used survival analysis to estimate the onset of GVHD after bone marrow transplant (BMT). We also plotted the survival graphs for the occurrence of GVHD for the whole population according to gender and indication for BMT. The log-rank test was employed to compare the equality of survivor function across these curves. A P-value of < 0.05 was considered statistically significant. The collected data were analyzed using the Stata Version 15.1 (© StataCorp, College Station, Texas, USA).

##  RESULTS

The mean age of the patients in the present study was 34.3 ± 11 years; it was higher in females compared to males (38.7 ± 7.2 vs 33.2 ± 11.6; P = 0.28). Totally, the study comprised 6 females and 24 males. The most common indications for BMT were acute lymphocytic leukemia (n = 7), acute myeloid leukemia (n = 7), and chronic myeloid leukemia (n = 6). The mean time to the diagnosis of oGVHD in the population was 232.8 days (95% CI: 153.6, 311.9) [Figure 1a]. Although the mean time to diagnosis of oGVHD was higher in males (254.4, 95% CI: 157.9, 351.0) compared to females (145.8, 95% CI: 129.1, 162.5), the difference was not statistically significant (P = 0.57) [Figure 1b]. The time to diagnosis was minimum for AML (141.0, 95% CI: 102.2, 179.8) followed by CML (174.6, 95% CI: 90.0, 259.2) [Figure 1c], but there was no significant difference in the curves (P = 0.16) [Figure 1c].

In order of frequency, the systemic involvement in these patients was as follows: oral cavity (66%), liver (56%), skin manifestations (49%), gastrointestinal tract (26%), musculoskeletal system (13%), lungs (11%), and penile changes (3%). About 59% of these patients had vitamin D deficiency, 39% had vitamin B12 deficiency, and 43% had prior cytomegalovirus reactivation. The patients were on the following systemic medications: oral antibiotics (75%), oral antivirals (66%), oral immunosuppressants (including steroids) (62%), oral antifungals (57%), and autologous serum (30%). It is worth mentioning that about 16% of these individuals also reported prior acute GVHD.

Most of the patients had normal bilateral anterior (n = 13, 43%) and posterior segment (n = 23, 77%) lesions on their first visit. The common findings at the first visit consisted of bilateral blepharitis (n = 5, 17%), meibomitis (n = 4, 13%), and conjunctival congestion (n = 3, 10%). During the first visit, we also recorded other anterior segment findings such as discharge, blepharospasm, conjunctival congestion, telangiectasia, and lid margin keratinization (seen in patients with chronic dry eye). At the first visit, bilateral uveitis was seen in one patient and unilateral uveitis in another. At 18 months, only 6 (20%) patients had normal bilateral anterior segment and 16 (53%) had bilateral normal posterior segment. Bilateral cataract was noted in one (3%) patient at the first visit; however, at 18 months, five (17%) patients had bilateral cataract and one patient had cataract in one eye. One patient had cataract surgery with intraocular lens implantation at the 18-month follow-up. Two patients had lid margin keratinization and blepharospasm; no other symptoms of cicatricial conjunctivitis were observed. Detailed slit-lamp findings of the anterior and posterior segments are described in Tables 1 and 2.

Table 3 presents the corneal findings. The proportion of individuals with bilateral clear corneas ranged from 13% to 23% up to 18 months. In our study, grade 1 (n = 17), grade 2 (n = 9), and grade 3 (n = 4) superficial punctate epithelial erosions (SPEEs) were seen at the first visit. However, SPEEs were seen in only 11 eyes at 18 months; all of these were grade 1 SPEEs. Bilateral filaments, tear film defects (tear film not uniformly formed over the cornea), keratitic precipitates, and tear film debris (solidification of mucin layer of the tear film) were other presentations in the study population. Certain clinical presentations and findings are shown in Figures 2a, 2b, 3, and 4.

Figure 5 presents the results of the OSDI questionnaire concerning the patients' right and left eyes. Most of the patients (41%) were graded as severe dry eye at the first visit and as moderate (50%) at 18 months. Figure 6 shows improvement in Lissamine corneal and conjunctival staining at 18-month follow-up. The results of the Schirmer test and TBUT are shown in Figures 7a and 7b, respectively. The prevalence of dry eye in our study population was 40% (24/60 eyes).

The patients were on the following medications: immunosuppressive drops (26/36, 72.2%), antibiotic drops (8/36, 22.2%), steroid drops (18/36, 50%), and lubricating drops (32/36, 88.9%). The immunosuppressive drops included cyclosporine 0.05%, fluorometholone 0.1%, loteprednol 0.5%, and tacrolimus 0.05%.

The findings related to patients who were followed-up at our center after two years of the initial visit are presented in Tables 1–3. Specifically, 12 patients attended the fifth visit, 8 patients the sixth visit, and 5 patients the seventh visit. The common findings after two years were posterior subcapsular cataract, PCIOLs, telangiectasia, blepharospasm, conjunctival congestion, grade 1 SPEEs, corneal filaments, and tear film debris. We also observed certain other complications such as meibomitis, blepharitis, follicular conjunctivitis, blepharospasm, lid margin keratinization, corneal and conjunctival punctate epithelial erosions, and filamentary keratitis due to dry eye. The cataract we noticed in some of the patients after two years might be due to prolonged steroid intake (either topical or systemic) or age-related changes.

##  DISCUSSION

The present longitudinal study describes the clinical features of oGVHD in patients who had undergone allogenic stem cell transplantation. We also followed these individuals to assess changes in their ophthalmic presentations over time. The most common part involved was the cornea, followed by the anterior and posterior segments. The common findings were grade 1 and grade 2 SPEEs, blepharitis, meibomitis, and conjunctival congestion. The systemic organs involved were oral cavity, liver, skin, and gastrointestinal tract. Most of these patients were on oral antibiotics, antivirals, and immunosuppressants. Some of long-term findings were cataract, PCIOLs, SPEEs, telangiectasia, blepharospasm, corneal filaments, and tear film debris.

In our study, the mean time to the diagnosis of oGVHD was 7.7 months. The time of onset of ocular manifestations varies across studies; some report a similar time as our study, whereas others have reported the late occurrence of oGVHD.^[13,19,20]^ For instance, Khan et al reported that the mean time of occurrence of oGVHD was 17.8 months, whereas Shikari et al found the mean time to be about 9.3 months.^[13,19]^ Although studies have shown the significant association of oGVHD with acute GVHD,^[13,14,21]^ in our study, only one-sixth of patients had a prior history of acute GVHD. As observed in our study and confirmed by other researchers, systemic involvement is reported in the majority of patients with oGVHD affecting the skin, oral cavity, and liver.^[12,19,22]^ On the other hand, the mean time to the diagnosis of oGVHD varies across different studies. The NIH Consensus Development Project has established diagnostic criteria for chronic GVHD in its 2005 and the subsequently revised 2015 report.^[15,23]^ However, Shikari et al argue that these criteria were developed by non-ophthalmologists.^[19]^ Indeed, the NIH working group also recognizes that patients are diagnosed late in the course of the disease and, thus, it is working on developing criteria for early diagnosis of chronic GVHD.^[24]^ If the diagnostic criteria are standardized and consistently implemented, it is likely that the variability in diagnosis and time of presentation may decrease.

The ocular manifestations of chronic GVHD may involve all layers of the eye, but the posterior segment is rarely involved.^[1]^ Furthermore, due to the immunological origin of this condition, the presenting complaints may be more general and include painful eye, dry eye, or redness of the eye rather than any specific symptoms.^[25]^ As seen in our study, grade 1 SPEEs were the most common presentation in these patients. Although the proportion varied over the next 18 months, patients still had features suggestive of grade 1 SPEEs. However, none of these patients developed grade 2 or grade 3 SPEEs at 18 months or later. These changes were also reflected in the clinical concerns of these patients. We also found that lissamine positivity had diminished at 18 months compared with baseline. Previous studies have also shown an association between corneal stain and progression of oGVHD.^[26,27]^ Corneal complications may lead to reduced vision and blindness.^[28,29]^ Some of these complications may be present even after the remission of systemic symptoms, and amniotic membrane keratoplasty or tectonic keratoplasty have been used to manage these patients.^[30,31]^

The other common findings in our study were meibomitis, blepharitis, and conjunctival congestion. Ogawa et al have also reported a high proportion of HSCT cases (48%) with meibomian gland dysfunction.^[32]^ Although the exact pathogenesis for meibomian gland dysfunction remains unclear, several hypotheses such as T-cell damage of the hair follicle bulges and keratinization have been suggested as potential pathogenic mechanisms.^[32,33,34,35]^ It has also been reported that meibomian gland dysfunction may be useful for early diagnosis of GVHD.^[32]^ The proportion of individuals with cataract increased during the follow-up period of three months in the present study. Cataract is a feature reported by others as well and may be associated with steroid use.^[1,7,36,37]^ However, we did not observe any case of glaucoma in our study population which may also be associated with the use of steroids.^[1,7,36,37]^

We used multiple methods to diagnose dry eye in the patients. Although Schirmer test is an important diagnostic tool, its reliability in diagnosis and management has been questioned by some authors.^[32,38]^ In our study, changes in the Schirmer score did not reflect changes in the ocular presentation of GVHD. The median TBUT in both eyes, however, was 5 s at baseline and later changed to 4 s at 18 months. The ODSI is another important questionnaire used to diagnose and measure the severity of dry eye disease.^[39]^ Our results showed that changes in OSDI reflected the clinician- and patient-reported changes in symptoms. Some studies have reported that, compared with Schirmer scores, changes in OSDI scores can better reflect changes in clinical signs and other symptom scales for patients with oGVHD.^[40,41,42,43]^ Thus, it is preferable to use multiple methods for the diagnosis and management of dry eye in patients with oGVHD.

Although we conducted a complete ophthalmologic evaluation, most of the patients continued the follow-up for only 18 months, with only a few continuing it beyond two years. It is highly possible that some of the presentations may occur after this time point, and we may have underestimated the long-term presentations of oGVHD. Furthermore, although we had taken a detailed history and the patients were being monitored, we may have underestimated the occurrence of acute GVHD in these individuals. However, the main purpose of our study was to investigate the longitudinal presentation of ocular symptoms and signs in patients with chronic GVHD.

Despite these limitations, our study provides useful data on the ocular manifestations of chronic GVHD over time. We found that the most common presentations initially were SPEEs (unilateral and bilateral), meibomitis, blepharitis, and conjunctival congestion. It appears that, thanks to clinical management, the proportion of grade 2 and grade 3 SPEEs and other inflammatory conditions decreased over the 18-month period. However, there was an increase in the proportion of unilateral and bilateral cataracts. In general, posterior segment manifestations were uncommon, suggesting that the ocular manifestations of chronic GVHD may change over time. It is important, therefore, to regularly monitor these patients so as to identify and manage both initial and late ocular manifestations of chronic GVHD.

### Financial Support and Sponsorship

None.

### Conflicts of Interest 

None.
